# Traumatic Bilateral Lumbosacral Jumped Facet Without Fracture in Childhood: Case Report and Systematic Review

**DOI:** 10.3390/jcm14176228

**Published:** 2025-09-03

**Authors:** Maria Ilaria Borruto, Michele Pomponi, Calogero Velluto, Achille Marciano, Luca Proietti, Laura Scaramuzzo

**Affiliations:** Department of Aging, Orthopaedic and Rheumatological Sciences, Fondazione Policlinico Universitario Agostino Gemelli IRCCS, 00168 Rome, Italy; maria.ilaria.borruto@gmail.com (M.I.B.); michele.pomponi01@icatt.it (M.P.); achille.marciano01@icatt.it (A.M.); luca.proietti@policlinicogemelli.it (L.P.); laura.scaramuzzo@policlinicogemelli.it (L.S.)

**Keywords:** pediatric spine trauma, jumped facet, lumbosacral dislocation, traumatic spondylolisthesis, facet joint, posterior fusion

## Abstract

**Background/Objectives:** Traumatic dislocation of the lumbosacral facet joints without associated fractures is exceedingly rare in the pediatric population. Due to the unique anatomical and biomechanical features of the pediatric spine, such injuries present diagnostic and therapeutic challenges. This study aims to describe a rare case of bilateral L5–S1 jumped facets without fracture in a 13-year-old boy and to review the existing literature on pediatric traumatic facet dislocations. **Methods:** We performed a systematic review according to PRISMA guidelines, searching PubMed, Embase, Scopus, and the Cochrane Library up to 16 January 2025. Keywords included “pediatric traumatic spondylolisthesis” and “pediatric traumatic facet joint”. Eligible studies reported traumatic lumbosacral or thoracolumbar facet dislocations in patients aged <18 years. In addition, we report the clinical course, surgical management, and outcome of a representative case from our institution. **Results:** The systematic review identified 14 pediatric cases across 11 studies. Most patients were male (71.4%), with high-energy trauma as the primary mechanism. The L5–S1 level was most frequently involved (57.1%). Neurological impairment was present in 57.1% of cases. All patients underwent surgical treatment, with posterior fixation being the most common approach. Our case involved bilateral L5–S1 jumped facets without fracture, successfully treated with open reduction and posterior fusion. Postoperative recovery was favorable, with neurological improvement. **Conclusions:** Traumatic bilateral facet dislocation without fracture is an extremely rare but serious condition in pediatric patients. Early recognition and surgical stabilization are essential to prevent permanent neurological damage. This study reinforces the importance of advanced imaging and prompt multidisciplinary management in optimizing outcomes.

## 1. Introduction

The facet joint is a key anatomical component of the spine due to its biomechanical role in facilitating vertebral articulation. These are diarthrodial joints composed of opposing articular cartilage surfaces enclosed by a ligamentous capsule, which ensures low-friction movement. Together with the intervertebral discs, the bilateral facet joints guide and constrain spinal motion, while also contributing significantly to load transmission thanks to their specific geometry and mechanical function [1].

Advanced imaging techniques are essential for detecting and characterizing facet joint injuries [2]. Among the traumatic pathologies involving this structure, traumatic lumbar spondylolisthesis—also referred to as traumatic lumbar locked facet syndrome—is a rare but severe injury. It involves an acute anterior displacement of one lumbar vertebra (from L1 to L5) relative to the subjacent segment, typically due to high-energy trauma. Unlike the more common degenerative forms of lumbar spondylolisthesis, the traumatic variant indicates a forceful mechanism sufficient to disrupt robust musculo-ligamentous stabilizers.

One classic example of such trauma is the so-called “suicide jumper’s fracture” [3], reflecting the high-energy nature of the underlying injury mechanism.

### 1.1. Epidemiology

Traumatic lumbar spondylolisthesis is a rare injury, typically resulting from high-energy trauma. It is most commonly observed in motor vehicle collisions or occupational accidents, such as in factory settings where the lumbar spine may be struck by heavy machinery. The condition predominantly affects males, with the peak incidence reported between 35 and 55 years of age [4].

Due to the deep location of the lumbar spine beneath thick musculature and the stabilizing role of large vertebral bodies and ligamentous structures, substantial kinetic energy is required to produce the degree of disruption seen in traumatic spondylolisthesis. As a result, its incidence remains significantly lower compared to other spinal trauma patterns.

### 1.2. Anatomy and Biomechanics

The posterior column of the spine includes the transverse processes, spinous processes, laminae, and posterior articular processes (zygapophyses). In the lumbar region, zygapophyseal joints are formed by the articulation of the inferior articular processes of a superior vertebra with the superior articular processes of the vertebra below.

The orientation of lumbar facet joints is critical in understanding the biomechanics of traumatic spondylolisthesis. Anteriorly, the facets are oriented more coronally, which allows resistance against lateral bending. Posteriorly, they shift to a more sagittal orientation, offering increased resistance to rotational forces. This anatomical configuration presents a “C” or “J” shape in the axial plane while appearing relatively flat in the coronal plane. In general, the more sagittal-oriented the facet joints, the less effective they are at preventing anterior translation, thus predisposing to instability.

Lumbar spine stability is further reinforced by multiple ligamentous structures, each with specific biomechanical roles. Flexion is resisted by the ligamentum flavum, supraspinous, interspinous, and posterior longitudinal ligaments; extension is limited by the anterior longitudinal ligament; and contralateral flexion is restricted by the intertransverse ligaments. Additionally, the iliolumbar ligaments offer resistance against anterior translation at the L5–S1 junction [4]. Muscular support is provided by the erector spinae, psoas major, and quadratus lumborum muscles, which contribute to dynamic stabilization of the lumbar spine.

In pediatric patients, the spine is more elastic due to shallower facet joints and more compliant ligamentous and capsular structures, rendering it more susceptible to certain types of injury patterns under stress.

### 1.3. Mechanism of Trauma

Lumbar spondylolisthesis can be categorized into several subtypes: dysplastic, isthmic, degenerative, traumatic, and pathologic. Among these, traumatic spondylolisthesis is the least common, primarily due to the deep location of the lumbar spine beneath the thick musculature of the back. A high-energy mechanism is typically required to induce this type of injury. A transverse process fracture in the lumbar spine is often a key clinical indicator that suggests the possibility of traumatic spondylolisthesis.

The primary mechanism for traumatic spondylolisthesis with bilateral facet dislocation is hyperflexion combined with varying degrees of distraction. In this scenario, the inferior articular facet of the superior vertebra is displaced anteriorly and becomes “locked” in front of the superior articular facet of the vertebra below. This hyperflexion mechanism may result in either pure facet dislocation or fracture-dislocation of the lumbar spine.

When facet joint disruption is unilateral, the injury mechanism often includes an additional rotational component. This can cause displacement of the superior and inferior articular processes due to injuries to the ligaments that normally stabilize the facet joints. Such injuries are typically observed at the junctions between more rigid and mobile parts of the spine, such as the thoracolumbar or lumbosacral junctions. The classic example of this injury mechanism is the “seatbelt injury,” which occurs during a motor vehicle accident when a seatbelt fails to secure the lower part of the spine while the upper part of the body is hyper-flexed and displaced anteriorly.

Although this injury mechanism is more prevalent in the cervical spine, where the anatomy and injury mechanisms are different, it does occur in the lumbar spine. The lumbar spine’s larger vertebrae and the vertical orientation of the facet joints contribute to the spine’s stability. In contrast, the more horizontally oriented facet joints at the lumbosacral junction are more susceptible to dislocation due to the increased mobility of the sacral region. This anatomical vulnerability, combined with the dynamics of hyperflexion and rotation, predisposes the L5S1 level to traumatic spondylolisthesis.

In the lumbar spine, the direction of displacement (anteroposterior versus lateral) often corresponds to the orientation of the facet joints. The more sagittal-oriented the facets, the greater the likelihood of lateral listhesis, while coronal facets predispose to anteroposterior displacement. Traumatic failure of the facet joints can be clearly visualized in imaging studies and often results in significant misalignment of the vertebral bodies, leading to spinal instability and potential neural compromise [5].

### 1.4. Radiological Findings

When the facet joint is disrupted, the normally posteriorly located inferior articular process shifts anteriorly. This movement is so pronounced that the inferior articular process may override the superior articular process, a condition referred to as a “perched facet.” In cases of more severe shear forces, the inferior articular process may move even further forward and become locked anteriorly to the superior articular process, a phenomenon known as a “locked facet.” This disruption of the facet joint leads to misalignment of the vertebral bodies, which in turn narrows the spinal canal and compromises the neural structures within.

These structural changes are easily detected through CT imaging with 3D reconstruction, as well as through an MRI. For visualizing trauma to the spinal cord or cauda equina, MRI is the preferred modality as it also highlights injuries to the paraspinal muscles. Traumatic strain can lead to muscle edema, which appears as hyperintense signal changes on STIR sequences. Key features in imaging include the loss of normal apposition at facet joints and an increased interspinous distance. Disc assessment is also crucial in all cases, as severe disc injuries may require fusion, even in the absence of an anterior slip.

Although some of these changes can be observed on initial trauma X-rays of the spine, CT scanning remains the diagnostic test of choice. Facet joint dislocations may present with a “reverse hamburger sign” (where the normal facet joint is likened to a hamburger), also known as the “double facet sign” or “naked facet sign.” In this case, the superior facet of the caudal vertebra is absent the normal opposing inferior facet of the vertebra above it. MRI is particularly useful for assessing injuries to the anterior and posterior longitudinal ligaments (PLL), ligamentum flavum, interspinous ligaments, and intervertebral discs, as well as for identifying any epidural hematomas [6,7].

The concept of traumatic lumbar spondylolisthesis was first described by Watson-Jones in the 1940s and later incorporated into classifications by Wiltse [5,8,9]. In 2019, Dimar JR 2nd proposed a new classification system for traumatic lumbar spondylolisthesis based on anatomical injury. This classification includes the following types:

Type 1: Unilateral or bilateral facet jump/dislocation;

Type 2: Unilateral or bilateral facet fracture;

Type 3: Acute unilateral or bilateral pars fracture;

Type 4: Acute fracture of a previously fused segment;

Type 5: Bilateral pedicle fracture;

Type 6: Complex fracture-dislocation with vertebral body involvement [5].

### 1.5. Clinical Presentation

Traumatic lumbar spondylolisthesis can lead to cauda equina syndrome in some cases. A significant number of patients initially present with low back pain, often without neurological deficits. Most injuries occurring in the lower lumbar region are low-grade anterolisthesis, and this, combined with the typically wide spinal canal at the L5–S1 level, may explain the absence of extensive neurologic deficits in many cases.

However, a high index of suspicion is essential during polytrauma evaluation, particularly when there are coexisting abdominal degloving injuries. In such cases, patients may present without obvious neurologic symptoms but with severe injuries to other systems, which may take precedence over immediate treatment for traumatic spondylolisthesis.

Although rare, cauda equina syndrome should always be ruled out. If present, immediate decompression is necessary. The primary goal in the management of polytrauma patients is to stabilize the traumatic spondylolisthesis as an emergency measure to prevent neurologic deterioration [5].

### 1.6. Management

Surgical intervention is the cornerstone of treatment for traumatic lumbar spondylolisthesis. Early reports in the literature highlight the failure of nonoperative management and non-fusion, leading to progression of the spondylolisthesis and secondary neurological impairment due to instability [5].

The timing of surgery is dictated by the presence or progression of neurologic deficits, but in an ideal scenario, the deformity should be stabilized as soon as possible. This approach is crucial because reduction becomes increasingly difficult over time [10].

For unilateral lumbar facet dislocation, the standard treatment typically involves open reduction and instrumented fusion. However, several variations of this approach have been suggested in the literature, including reduction with posterior instrumented fusion alone, posterior instrumented fusion combined with anterior interbody fusion, or different levels of fusion depending on the specific case [6].

In general, most experts do not recommend decompressive laminectomy in the absence of neurologic symptoms, as this may lead to further instability. Partial facetectomy may be utilized to facilitate reduction, although it’s important to note that intact apophyseal joints can still act as a barrier against redislocation in the future [11].

Circumferential fusion is commonly recommended, particularly when there is disc disruption and instability [5]. The degree of instrumentation and arthrodesis may be influenced by damage to the posterior tension band. Dislocation reduction is typically achieved by removing the superior L5 articular facet and performing limited lateral osteotomies [12].

## 2. Materials and Methods

### 2.1. Study Setting and Search Strategy

**Figure 1:**
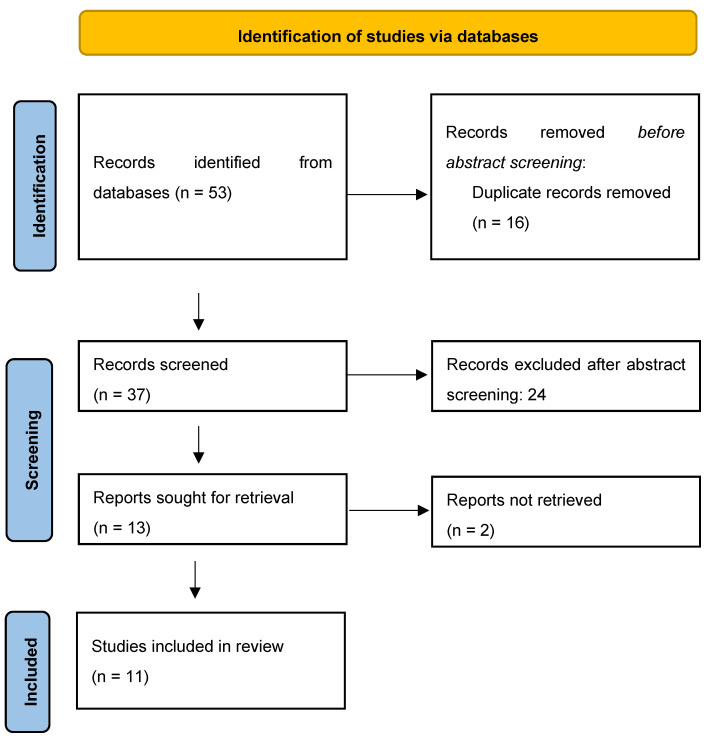
PRISMA flow-chart.

In the present study, a systematic literature review according to the Preferred Reporting Items for Systematic Reviews and Meta-Analyses (PRISMA) guidelines was performed (Figure 1). The review was not prospectively registered in any database, as no registration was performed for this study. MEDLINE via PubMed and Embase, Scopus, Cochrane Library database were searched using the keywords: “pediatric traumatic spondylolisthesis”, “pediatric traumatic facet joint” and their MeSH terms in any possible combinations using the logical operators “AND” and “OR”. The reference lists of relevant studies were forward screened to identify other studies of interest. The search was reiterated until 16 January 2025.

### 2.2. Inclusion and Exclusion Criteria

In this review, the full-text articles describing pediatric traumatic spondylolisthesis due to facet joint disruption were considered eligible. Only articles written in English were included. No date limits were set for publication. Expert opinions, studies on animals, unpublished reports, in vitro investigations, case reports, letters to the editor, abstracts from scientific meetings, and book chapters were excluded from review.

### 2.3. Data Extraction and Analysis

Two independent authors (M.I. and M.P.) searched and collected data from the included studies. Any discordances were solved by consensus with a third author (L.S.). The following data were extracted: demographic features, diagnosis, comorbidities, possible complications, clinical outcomes, and follow-up. Risk of bias assessments and quality assessments of included studies was checked using Cochrane risk of bias tool. Numbers software (Apple Inc., Cupertino, CA, USA) was used to tabulate the obtained data. Categorical variables are presented as frequency and percentages. Continuous variables are presented as means and standard deviation. Only one decimal digit was reported and was rounded up.

## 3. Case Report

A 13-year-old boy was admitted to our emergency department after a second-floor fall. On admission, he was intubated and sedated with isochoric and isocyclic pupils. Review of the cranial CT scan reveals left hemispheric edema with compression on the ipsilateral ventricular system structures, midline shift, and early uncal herniation.

**Figure 2:**
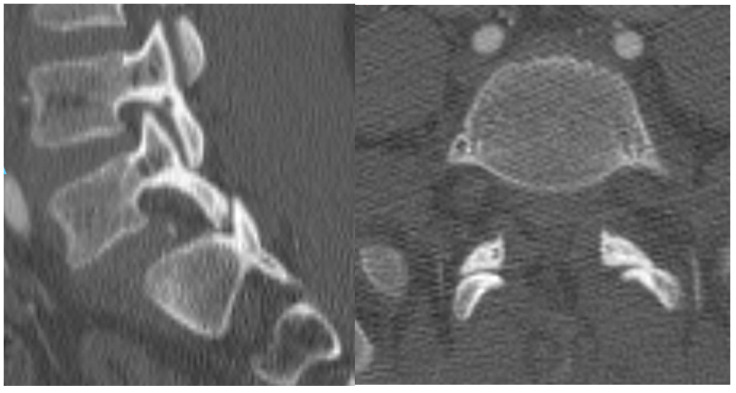
CT scan showing bilateral jumped facet.

A computed tomography (CT) scan of the lumbar spine revealed bilateral jumped facets at L5–S1 without fractures, resulting in a Grade-II anterolisthesis of L5 on S1 and canal narrowing (Figure 2).

The case indicates a surgical need for cranial decompression. During the procedure, it was decided not to reposition the skull cap.

**Figure 3:**
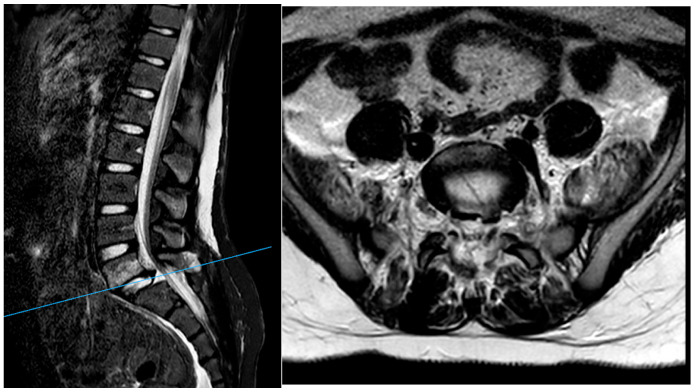
MRI showing the rupture of the L5–S1 disc with disruption of the anterior and posterior longitudinal ligaments and the interspinous and supraspinous ligaments.

After surgery, MRI of the lumbar spine (Figure 3) further identified rupture of the L5–S1 disc, with disruption of the anterior and posterior longitudinal ligaments as well as the interspinous and supraspinous ligaments from L4 to S1.

**Figure 4:**
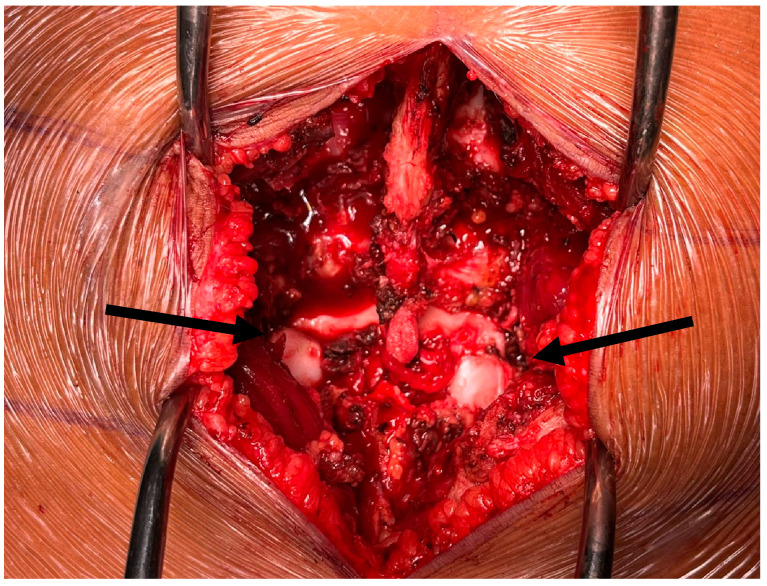
Intraoperative findings: disruption of the facet joint. It is visible the cartilage surface of the inferior facet joint.

The patient was taken to the operating room the following morning. A large paraspinous muscle hematoma with posterior tension band disruption was encountered (Figure 4).

**Figure 5:**
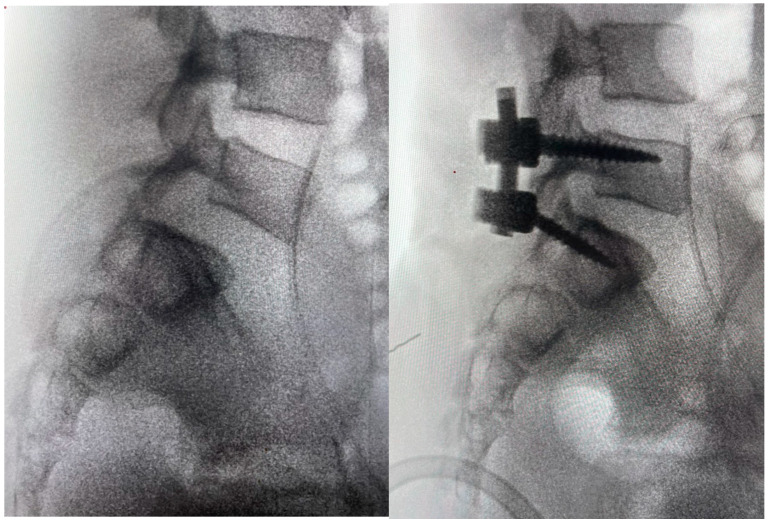
Intraoperative fluoroscopic image before and after surgery, showing the reduction.

Reduction maneuvers were attempted, and the right superior S1 facets were removed with a high-speed drill until the L5 inferior facets disengaged, easily resulting in listhesis reduction and spinal realignment. Of note, the reduction and realignment after disengagement of the L5 inferior facets was immediate but not full, requiring further manipulations, such as a cantilever technique. Segmental pedicle screw fixation with posterolateral arthrodesis was performed from L5 to S1 with morselized bone autograft (Figure 5). In our reduction, we removed the tip of the inferior facet of L5 and levered the inferior facet of L5 over the facet of S1. Then we performed a posterolateral fusion, using screw and autologous bone graft. We decided not to decompress in order to prevent further instability.

At 6-month follow-up, after recovery from the traumatic brain injury, the patient presented with no neurological deficits.

## 4. Results

After conducting an electronic literature search, 11 articles were retrieved and reviewed for eligibility. These studies were screened by title and abstract after removing duplicates. All 11 articles were deemed suitable for full-text revision, and relevant clinical, surgical, and radiological data were extracted and analyzed. The included studies, classified as Level of Evidence (LoE) IV, collectively reported 14 pediatric cases of traumatic lumbosacral or thoracolumbar facet dislocations, which are detailed in Table 1 and derived from the original case reports found in the literature [13,14,15,16,17,18,19,20,21,22,23].

### 4.1. Demographic and Clinical Characteristics

The study reviewed 14 pediatric cases (Table 1) of traumatic facet dislocations, with patient ages ranging from 4 to 18 years (mean age: 10.8 years). The majority of patients were male (10/14; 71.4%), consistent with previous studies showing that boys are at higher risk for high-energy trauma due to greater exposure to physical activity and environmental hazards [24]. The mechanisms of injury were primarily high-energy, with three patients (21.4%) being trapped under collapsed structures, two (14.3%) falling from height, and others involved in vehicular or crush-related trauma. Our own case, presented in this report, involved a 13-year-old boy with bilateral L5–S1 facet dislocation without vertebral fracture following a hyperflexion injury, representing an isolated posterior element injury in a pediatric patient.

### 4.2. Injury Patterns and Neurological Status

The L5–S1 junction was the most frequently affected level (8/14; 57.1%), followed by L4–L5 (2/14), L1–L2 (2/14), and one case each at L2–L4 and L2–L3–L4. These findings emphasize the vulnerability of the lumbosacral junction to translational forces in younger patients, despite its anatomical stability [25]. Associated injuries were noted in more than half of the cases, including sacral fractures, transverse process fractures, perineal trauma, pneumothorax, and long bone injuries. Neurological status at admission, as assessed by the ASIA Impairment Scale, showed that six patients (42.9%) were classified as ASIA A, with others presenting as ASIA B (1), ASIA C (2), ASIA D (3), and ASIA E (2). One representative case involved a 13-year-old patient with an initial ASIA C score, who achieved partial neurological recovery to ASIA D at follow-up, with independent ambulation and residual distal weakness.

### 4.3. Surgical Management and Clinical Outcomes

All patients underwent surgery due to spinal instability and failure of conservative management. The most common surgical approach was posterior fixation (9/14; 64.3%), with a combined anterior-posterior approach in four cases (28.6%). Surgical techniques included pedicle screw-rod constructs, vertebrectomy with cage insertion, and in one case, a modified Luque frame with sublaminar wires [22]. Our institutional case involved posterior spinal fusion using pedicle screws and autologous iliac crest graft.

Postoperative outcomes revealed neurological improvement in three patients (21.4%), while six patients (42.9%) with complete deficits at presentation showed no change. Four patients (28.6%) maintained their intact neurological status pre- and postoperatively. Unfortunately, one patient died due to complications from multisystem trauma [15]. These results are consistent with previous studies, indicating that the degree of initial spinal cord injury is a critical prognostic factor [26].

### 4.4. Radiological and Functional Outcomes

Follow-up imaging confirmed proper realignment and evidence of spinal fusion in most cases. However, some patients experienced persistent deficits, such as foot drop or sensory changes, while others regained full functional independence. The incidence of posterior spondyloptosis or severe retrolisthesis was noted in three patients (21.4%), which aligns with prior recommendations advocating for extensive posterior instrumentation in cases with three-column ligamentous disruption [21,27]. MRI proved to be an essential tool in surgical planning, especially given the potential for occult soft tissue injuries in younger children [28].

In summary, the data from this case series, combined with our institutional experience, support the importance of early surgical stabilization in pediatric traumatic facet dislocations. These findings also underscore the role of hyperflexion mechanisms and reduced lumbar lordosis in facilitating sagittal plane instability at the lumbosacral junction [29].

## 5. Discussion

This study examined 14 pediatric cases of traumatic lumbosacral or thoracolumbar facet dislocations, focusing on demographic, clinical, and surgical outcomes. The majority of patients were male (71.4%), consistent with previous findings showing a higher risk of traumatic injuries in boys due to increased physical activity and exposure to environmental hazards. The most common injury mechanisms were high-energy trauma, such as being buried under collapsed structures, falls from height, and vehicular or crush accidents.

In terms of injury distribution, the L5–S1 junction was the most commonly affected level, highlighting the vulnerability of this region to translational forces, even in younger patients. This is in line with previous reports that identified L5–S1 as the predominant site for traumatic facet dislocation in pediatric populations [29,30]. Notably, a significant proportion of patients (42.9%) presented with severe neurological deficits (ASIA A), underscoring the potential for serious spinal cord involvement. However, some cases showed neurological improvement, particularly those with partial deficits at presentation, which is consistent with prior literature, suggesting that incomplete neurological injuries have a greater potential for recovery [17].

Surgical intervention was required for all patients due to the degree of instability and failure of conservative management. The majority of cases were treated with posterior fusion (64.3%), while combined anterior–posterior fusion was reserved for more complex injuries. These surgical strategies mirror those described in both pediatric and adult series of high-grade traumatic dislocations, including spondyloptosis, where stabilization is essential for preventing further neurological compromise [17]. Postoperative outcomes in our series demonstrated variable neurological recovery; patients with incomplete deficits fared better, while those with complete injuries at presentation generally experienced minimal change, reflecting the persistent prognostic impact of initial spinal cord damage. Radiological follow-up confirmed appropriate alignment and fusion in most cases, although some residual deficits, such as foot drop, were noted, a finding also reported in isolated pediatric case reports [15,29].

MRI proved crucial in surgical planning, allowing for detailed assessment of soft tissue and ligamentous injuries, as emphasized by previous authors [17,30]. Its role in preoperative evaluation is particularly relevant in pediatric patients, in whom subtle ligamentous disruption may not be evident on plain radiographs or CT.

Overall, our findings reinforce the importance of early surgical stabilization to prevent further neurological deterioration and optimize outcomes in high-risk pediatric patients with severe facet dislocations. Moreover, the literature suggests that long-term follow-up is essential to monitor spinal growth and fusion integrity, given the potential for late complications or deformity progression in skeletally immature patients [13].

Further studies will be needed to evaluate whether specific lumbosacral alignment parameters are altered following such traumatic injuries, ideally employing less invasive assessment methods [31].

This systematic review has several limitations that should be acknowledged. First, due to the rarity of pediatric traumatic facet dislocations, the available evidence is limited to isolated case reports and small case series, which restricts the generalizability of the findings. Second, publication bias may have influenced the results, as unpublished cases or studies with negative outcomes were not accessible and therefore could not be included. Third, there was considerable heterogeneity among the included studies, both in terms of clinical evaluation and outcome definitions, which complicates direct comparisons across cases. In addition, the quality of the available reports varied, with many lacking standardized reporting of clinical and radiological outcomes. Finally, long-term follow-up data were rarely provided, limiting the ability to assess the durability of surgical results and the impact on spinal growth and function in the pediatric population.

## 6. Conclusions

This study supports early surgical stabilization in pediatric patients with traumatic lumbar facet dislocations. Appropriate surgical intervention can improve spinal alignment and neurological function, although the severity of the initial spinal cord injury remains the main determinant of long-term outcomes. Future research should refine surgical techniques for pediatric high-energy trauma, explore adjunctive therapies to enhance neurological recovery, and emphasize the role of advanced imaging in preoperative planning.

## Figures and Tables

**Table 1:** Reported cases of traumatic jumped facet injuries in the pediatric population.

Name, Year	Sex	Age	Trauma	Other Lesion	Level	Approach	Treatment	ASIA	ASIA Post	Treatment Outcome
Vetter SY, 2015 [13]	M	12	buried beneath a wall	open fracture left distal fibula, epiphysiolysis distal tibia.	L5–S1	Posterior	closed reduction + percutaneo viti e barre	E	E	no deficit
Yamaki VN, 2018 [14]	F	4	buried beneath a wall		L5–S1	ant + post		D	D	able to walk independently, despite a persistent left foot drop
Mishra A, 2015 [15]	M	16	fall from height	bilat pneumothorax	T4–5	posterior	T3–T5 laminectomy and T3–T6 PSF	A	A	no improvement
Mishra A, 2015 [15]	M	12	crushing accident		L4–L5	posterior	L2–L5 PSF pedicle screw fixation	A	A	no improvement and development lumbar ernia
Mishra A, 2015 [15]	M	18	fall from height	T9 compression fracture	L1–L2	post + ant	L2 corpectomy + L3–L4 PSF	A	A	death
Esteves A, 2024 [16]	M	5	direct trauma to the lumbar spine (heavy object)	with L5 isthmic fractures and a sacral fracture	L5–S1	posterior	open reduction of the L5–S1 facet joint dislocation associated with posterior lumbopelvic fixation (L4-iliac)	E	E	no neurological deficits
Villarreal-Arroyo M, 2011 [17]	F	8	traumatic	none	L5–S1	posterolateral	pedicle scree fixation L4-S1	E	E	no neurologigal deficit
Chandrashekhara SH 2011 [18]	M	10	fell from a running truck and was run over by the tractor-plough a	fracture of L3, disruption of articular facets	L4–L5	Posterior	L2, L3, L4, L5 pedicle screw and rod fixation	A	B	paraplegia mild improvement
Chandrashekhara SH 2011 [18]	M	16	traumatic motor vehicle crash		L2–L3–L4	Post + ant	vertebrectomy L3 + posterior L1–L5	A	A	
Yang X, 2015 [19]	F	11	crushed under a collapsed beam	transverse process fracture (L1–L5 in right)	L5–S1	posterolateral	L5–S1 laminectomy, reduction, posterolateral fusion	D	E	No neurological deficit
Rodrigues LM, 2015 [20]	M	15	buried beneath a wall		L5–S1	post + ante	pedicle fixation and an anterior cage	C	D	PRE: bilaterally decreased muscle strength was observed POST: significant recovery of muscle strength in the lower limbs
Verhelst L, 2009 [21]	M	6	hit by a tractor	perineal breach, several hepatic lacerations, Morel-Lavalle’e lesion over the left hip and gluteal area	L5–S1	posterior	L3-S2 pedicle screw fixation and posterolateral grafting.	A	A	complete loss of perineal sensation and loss of anal sphincter tone were found
M Yazici, 1999 [22]	F	6	traumatic		L1–L2	Posterior	modified Luque frame with sublaminar wires.	C	E	pre: incomplete paraplegia post: no neurologic deficit
H Abdel-Fattah, 1990 [23]	M	18	traumatic		L4–L5	Posterior	Open reduction and internal fixation with a sacral rod and two Harrington rods	E	E	neurologically intact

## Data Availability

The original contributions presented in this study are included in the article. Further inquiries can be directed to the corresponding author(s).
